# Optimizing the Acceptability, Adherence, and Inclusiveness of the COVID Radar Surveillance App: Qualitative Study Using Focus Groups, Thematic Content Analysis, and Usability Testing

**DOI:** 10.2196/36003

**Published:** 2022-09-09

**Authors:** Bas Splinter, Nicholas H Saadah, Niels H Chavannes, Jessica C Kiefte-de Jong, Jiska J Aardoom

**Affiliations:** 1 Department of Public Health and Primary Care Leiden University Medical Center Leiden Netherlands; 2 National eHealth Living Lab Leiden Netherlands

**Keywords:** COVID-19, corona, eHealth, self-report, mobile app, track-and-trace strategies, population surveillance, citizen science, usability, mobile phone

## Abstract

**Background:**

The COVID Radar app was developed as a population-based surveillance instrument to identify at-risk populations and regions in response to the COVID-19 pandemic. The app boasts of >8.5 million completed questionnaires, with >280,000 unique users. Although the COVID Radar app is a valid tool for population-level surveillance, high user engagement is critical to the success of the COVID Radar app in maintaining validity.

**Objective:**

This study aimed to identify optimization targets of the COVID Radar app to improve its acceptability, adherence, and inclusiveness.

**Methods:**

The main component of the COVID Radar app is a self-report questionnaire that assesses COVID-19 symptoms and social distancing behaviors. A total of 3 qualitative substudies were conducted. First, 3 semistructured focus group interviews with end users (N=14) of the app were conducted to gather information on user experiences. The output was transcribed and thematically coded using the framework method. Second, a similar qualitative thematic analysis was conducted on 1080 end-user emails. Third, usability testing was conducted in one-on-one sessions with 4 individuals with low literacy levels.

**Results:**

All 3 substudies identified optimization targets in terms of design and content. The results of substudy 1 showed that the participants generally evaluated the app positively. They reported the app to be user-friendly and were satisfied with its design and functionalities. Participants’ main motivation to use the app was to contribute to science. Participants suggested adding motivational tools to stimulate user engagement. A larger national publicity campaign for the app was considered potentially helpful for increasing the user population. In-app updates informing users about the project and its outputs motivated users to continue using the app. Feedback on the self-report questionnaire, stemming from substudies 1 and 2, mostly concerned the content and phrasing of the questions. Furthermore, the section of the app allowing users to compare their symptoms and behaviors to those of their peers was found to be suboptimal because of difficulties in interpreting the figures presented in the app. Finally, the output of substudy 3 resulted in recommendations primarily related to simplification of the text to render it more accessible and comprehensible for individuals with low literacy levels.

**Conclusions:**

The convenience of app use, enabling personal adjustments of the app experience, and considering motivational factors for continued app use (ie, altruism and collectivism) were found to be crucial to procuring and maintaining a population of active users of the COVID Radar app. Further, there seems to be a need to increase the accessibility of public health tools for individuals with low literacy levels. These results can be used to improve the this and future public health apps and improve the representativeness of their user populations and user engagement, ultimately increasing the validity of the tools.

## Introduction

### Background

Since December 2019, the world has been battling the SARS-CoV-2 (COVID-19). In the Netherlands specifically, the first case was identified in February 2020 and has resulted, to date, in >2.6 million confirmed cases and 19,000 deaths [1]. Track-and-trace strategies and quarantine measures to limit social contact and social distancing have been widely used to prevent the transmission of the virus [2]. However, a delay between the appearance of symptoms and confirmed test results increase lead times during an outbreak. Continuous population-based monitoring of COVID-19–related symptoms and social distancing behaviors may aid in the estimation of risk of COVID-19 cases at a regional level, allowing local governments to intervene at an earlier stage [3].

Since the start of the pandemic, mobile apps have been implemented for a variety of goals such as risk assessment and decision-making as well as self-management and self-monitoring of symptoms [4]. Symptom-monitoring apps based on self-reporting show promising results in terms of predicting local COVID-19 spread using symptom-based tracking in the United Kingdom and United States [5,6]. Menni et al [5] found that a prediction model based on an app-based symptom tracker could predict a positive COVID-19 test with a sensitivity of 65% and a specificity of 78% [5]. Other studies have shown that population-wide self-reported data collected by means of a mobile app could identify 75% of the regions with the highest COVID-19 incidence according to governmental test data [6]. Finally, a recent study showed that longitudinal self-reported data on health, behavior, and demographics, as collected with web and mobile apps, could be adequately used in building prediction models to identify potential positive COVID-19 cases [7]. The above literature highlights the utility of self-reported data in population-based COVID-19 tracking and subsequent prediction of COVID-19 hot spots, which can ultimately aid in the response to the COVID-19 pandemic.

In the Netherlands, a smartphone-based surveillance app, the COVID Radar app, was developed. The goal of the app was to predict topographical future COVID hot spots with the help of self-reporting of COVID symptoms, enabling the possibility for local or national governments to intervene, for instance, by informing the public or implementing local restrictions. The app enables frequent and anonymous voluntary self-reporting of COVID-19–related symptoms and behaviors (users are asked only for their postcode, age range, sex, and profession). The app was developed during the first COVID-19 wave in the Netherlands in the spring of 2020. Among other functionalities, the app contains a short monitoring questionnaire (≥20 questions) asking users to self-report their COVID-19–related symptoms, social distancing behaviors, COVID-19 status, and vaccination status. The app additionally provides users feedback on their reported social distancing behavior (eg, number of people encountered within 1.5 meters and hours spent outside the house) and symptoms by enabling them to compare these to the mean values nationwide as well as within their specific geographic region. Since the launch of the app in April 2020, it boasts of >8.5 million completed questionnaires, with >280,000 unique users, of whom >13,000 completed the questionnaire at least twice a week on average. The COVID Radar app can be considered a citizen science project, as it involves the public in the scientific processes, in this case the collection of large-volume longitudinal symptom and social distancing data across the Netherlands [8].

Previous research has shown that the COVID Radar app is a useful and valid tool for population-level surveillance and potentially for the prediction of local COVID-19 hot spots [3]. More specifically, self-reported positive test results reported via the app closely matched government-reported case counts. In addition, there were clear associations between self-reported COVID-19 symptoms and positive test results. With respect to behavioral measures, a clear association was found between self-reported positive test results and above-average risk behaviors in terms of social distancing (eg, having had more visitors in one’s home or having had more people within 1.5 meters) in the days leading up to a test. Importantly, the identified associations among symptoms, social distancing behaviors, and test results were most pronounced in areas with high user engagement. Hence, high user engagement throughout different regions of a country seems critical to the success of any predictive model using self-reported symptoms and behavior. Stimulating and increasing user engagement by citizens in population-based surveillance apps such as the COVID Radar remains challenging.

The Netherlands Organization of Health Research and Development funded the COVID Radar project in which a multidisciplinary team aimed to develop a mobility- and behavior-based early warning system after the first wave of COVID-19 in the Netherlands. The methods in this project combine unique real-time spatial summaries per 4-digit postal code area of symptoms and high-risk behavior from the population surveillance data of the COVID Radar app at the Leiden University Medical Center (LUMC), with aggregated historic mobility information provided by mobile telecommunications data. One of the objectives of the project is to optimize the COVID Radar app and enrich the population surveillance syndrome and behavior data. The goal of this optimization of the COVID Radar app and survey is to ultimately improve the acceptability, adherence, and inclusiveness of the app and to learn lessons for improvement of future population-level health-related apps. This can subsequently support further national upscaling and use of the COVID Radar, as well as improve the representativeness of its user population. Furthermore, such targets can be subsequently used as inputs when designing national surveillance self-report data collection apps.

### Objectives

The main aim of this study was to identify the optimization targets of the COVID Radar app to ultimately improve the acceptability, adherence, and inclusiveness of the app. We aimed to identify optimization targets by conducting 3 qualitative substudies: (1) gathering and analyzing in-depth information on user experiences by means of semistructured focus group interviews with end users of the COVID Radar app, (2) analyzing all received end-user emails that were sent to the COVID Radar project team since the launch of the app, and (3) review by language experts and usability testing of the COVID Radar app in individuals with low literacy levels.

## Methods

### Design

All 3 substudies were qualitative research investigations. The first substudy consisted of 3 semistructured focus group interviews with COVID Radar end users. The second substudy comprised a qualitative analysis of all emails sent by the end users to the COVID Radar project team. The third substudy involved expert reviews and individual usability test sessions with individuals with low literacy levels. More details on the methods of each of these substudies can be found below.

### COVID Radar App

The COVID Radar app was freely available for iOS and Android systems and could be downloaded from both the Apple App Store and Google Play Store between April 2, 2020, and February 28, 2022. Upon its release, a brief publicity campaign was launched. First-time users are asked to register by providing information about (1) their gender (male, female, other, or not specified), (2) their age (10-year intervals from 0 to 80 and a ≥80 category), (3) their occupation (education, health care, catering industry, or other occupations with high risk of human contact within 1.5 meters), and (4) the 4 digits of their postal code.

**Figure 1:**
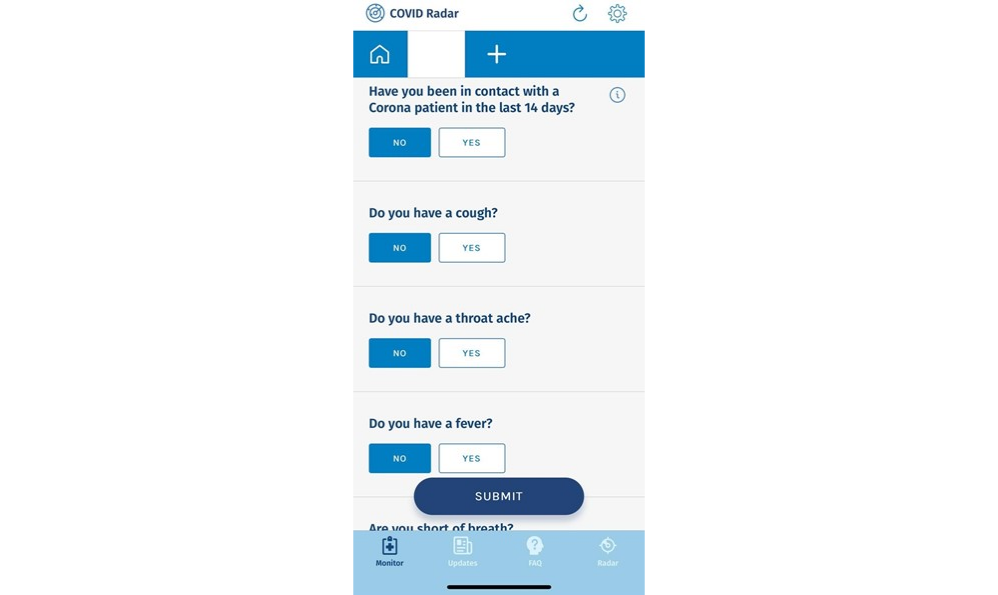
Screenshot of the monitoring section, presenting part of the self-report monitoring questionnaire.

The app consists of 4 sections. The first section contains the self-report monitoring questionnaire. A screenshot of the questionnaire is presented in Figure 1. The questionnaire is dynamic, meaning that the number and content of questions can be updated as needed based on new relevant insights related to the surveillance of the coronavirus. For example, questions about vaccination status were added as soon as the Dutch vaccination program started. The final questionnaire contained a total of 23 questions. The full version of the current questionnaire is presented in Multimedia Appendix 1. A total of 11 questions assess COVID-19 symptoms (eg, coughing, fever, and sore throat), and 8 questions assess social distancing behaviors, including among others, the number of human contacts within 1.5 meters, whether one has been in contact with a patient with COVID-19 infection in the last 14 days, how many visitors one had received on a particular day, whether the participant was ever tested positive (yes or no), whether the participant was tested in the last 2 weeks (no, have not been tested; have been tested—result was that I did not have the coronavirus; have been tested, result was that I did have the coronavirus), and a question about COVID vaccine status. Users receive a push message every other day to remind them to fill in the questionnaire, regardless of whether the user has already done so.

The second section of the COVID Radar app is an update section. This section is used to inform users on changes in the self-report questionnaire or to inform them about recent results of analyses of COVID Radar data.

The third section contains a frequently asked questions (FAQs) section, where users can find information on numerous topics related to information about the app (eg, the goal of the app), data protection (eg, which data are gathered exactly, and why will the data be saved for a period of 5 years?), contact information, and instructions on how to complete the questionnaire (eg, how to report symptoms when having asthma?).

**Figure 2:**
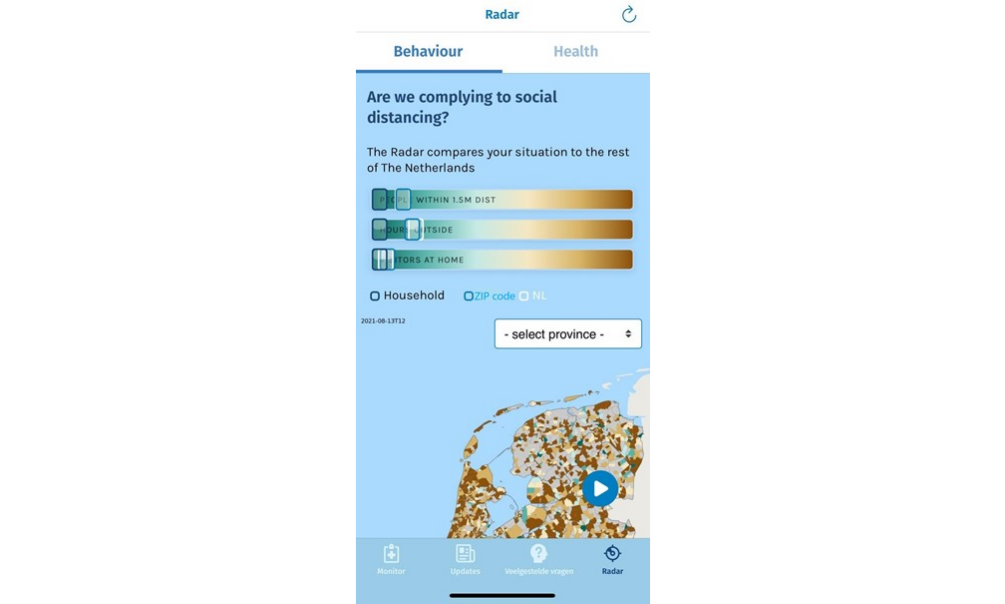
A screenshot of the Radar section, presenting a map with the mean symptoms per geographic region, as well as sliders providing feedback on the prevalence of symptoms compared with other users.

**Figure 3:**
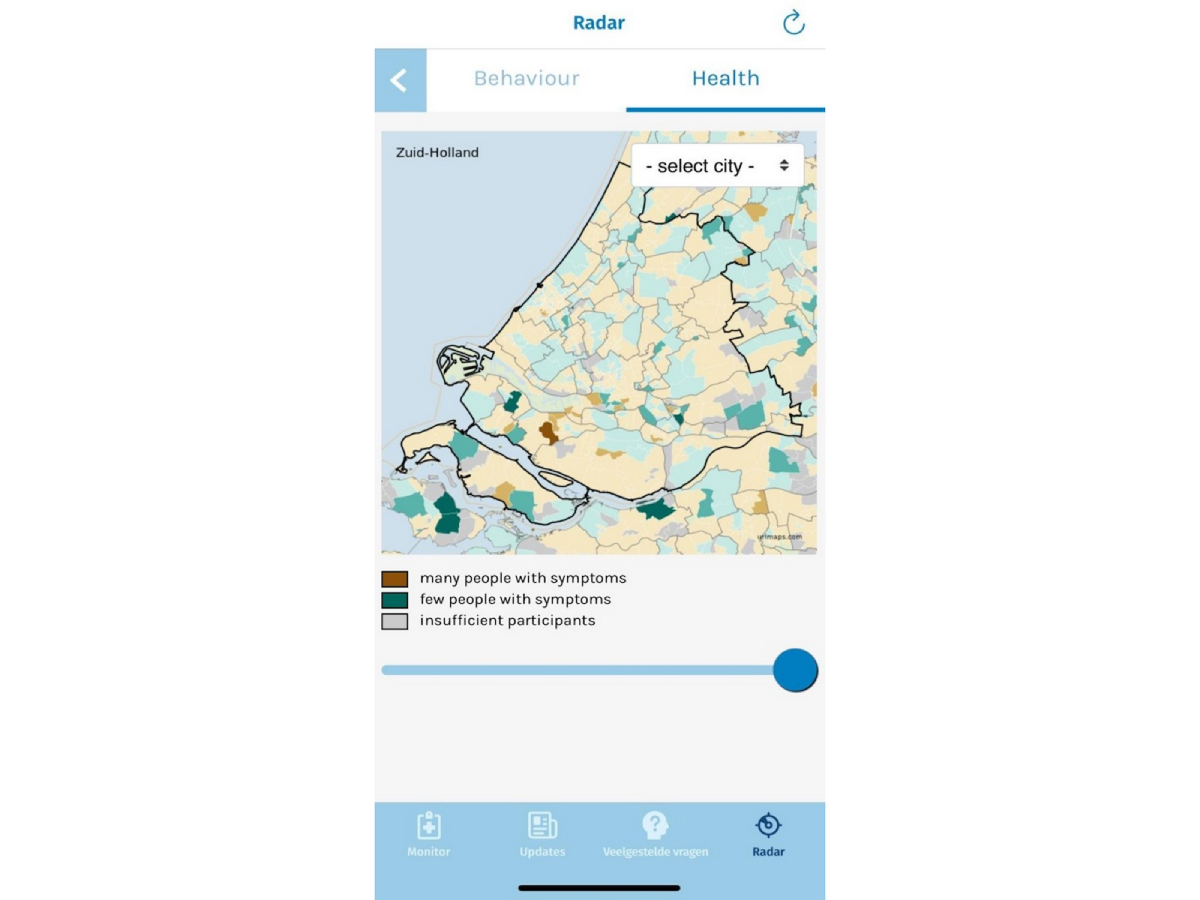
A screenshot of the Radar section, presenting a map with mean social distancing behaviors per geographic region, as well as sliders providing feedback on social distancing behavior in comparison with other users.

The fourth and final section is the Radar section, where feedback on user-reported symptoms and social distancing behavior can be observed. More specifically, app users can compare their self-reported social distancing behaviors (eg, the number of people spoken to within 1.5 meters) and symptoms to those of other users in their local region as well as the country’s app users as a whole. The feedback regarding social distancing behaviors is provided using sliders, colored from green to brown, with green representing *safe* behavior and red representing relatively *risky* behavior. A screenshot is provided in Figure 2. Figure 3 shows the mean prevalence of symptoms per geographic region, categorized as brown (many people with symptoms), green (few people with symptoms), and gray (insufficient participants to adequately measure the prevalence).

### Substudy 1: Focus Group Interviews With End Users

#### Procedure

COVID Radar app users were notified of the upcoming focus groups and invited to participate via an update in the update section of the app. The invitation contained a brief summary of the study aims and corresponding procedures. For participating in one of the focus groups, an individual had to be able to read, speak, and understand Dutch. Participants could indicate their interest in participating by sending an email to the research team. A group of 50 potential participants was randomly chosen from among the responses, who were subsequently asked to provide their availability for the upcoming focus groups. A total of 3 focus groups were scheduled to accommodate varying schedules. Because of possible technical issues, the focus groups were conducted with smaller groups than the offline focus group, as recommended by previous research [9,10]. A total of 5 available participants were chosen at random for each focus group.

Two weeks before the focus group would take place, participants received an information letter by email containing all study details and corresponding focus group procedures. The email also contained an informed consent form. Individuals were instructed to complete the form digitally or manually (ie, print, sign, and scan) and to return the completed form to the researchers by email. Participants received a €20 (US $24) gift card for their participation in the interviews.

#### Focus Group Interviews

A semistructured interview protocol was developed to serve as a guideline for the discussion of relevant topics during the focus group interviews. The interview protocol comprised open questions aimed at gathering participants’ experiences and opinions with respect to 7 topics: (1) general opinion of the app, (2) suggestions for improvement of the app, (3) feedback on the self-report monitoring questionnaire, (4) feedback on the update section, (5) feedback on the FAQs section, (6) feedback on the Radar section, and (7) feedback on the push notifications.

A total of 3 focus groups were conducted in May 2021. All focus group interviews were web-based, conducted via Zoom (Zoom Video Communications) with the audio being recorded. The interviews were moderated by BS. NHS and JJA were present as backup moderators and for support in case of technical problems. The duration of each focus group was approximately 60 minutes.

Audio recordings were transcribed literally. The names of participants were replaced with participant numbers to preserve their anonymity. Subsequently, the transcripts were thematically coded using the framework method [11]. The framework is a qualitative content analysis method. It was used to identify commonalities and differences in qualitative data before exploring the coherence of the data, focusing on finding explanatory conclusions per theme. The framework method works in different stages: (1) transcription, (2) familiarization with transcript, (3) coding, (4) developing an analytical framework, (5) applying the analytical framework, (6) charting data into the framework matrix, and (7) interpreting the data. During the process, it was possible to go back and forth between the different stages. This dynamic approach enabled the addition of new codes during the analysis, thereby creating an opportunity to react to unexpected findings and a broad spectrum of input on themes. This method is often used to analyze semistructured interview data. The identified themes were (1) general opinion of the app, (2) motivation for use, (3) publicity of the app and enlarging the user population, (4) self-report monitoring questionnaire, (5) in-app updates, (6) FAQs, (7) Radar section, and (8) push notifications. Coding was performed by BS and checked for reliability by JS by checking random samples of the coded transcripts. Coding was performed using Atlas.ti software (version 7.5.18). Data saturation was reached after conducting 3 focus group interviews.

### Substudy 2: Email Box Analysis

The COVID Radar team offered the possibility of app users sending feedback to a general email address. This email address was published in the FAQs section under the contact information. The goal was to enable the research team to subsequently optimize the app or handle technical errors as soon as possible. A total of 1080 emails received by the COVID Radar team between May 2020 and June 2021 were exported into a text document and subsequently uploaded to Atlas.ti software (version 7.5.18). The codebook designed for substudy 1 was used for thematic coding of the email feedback.

### Substudy 3: Review by Language Experts and Usability Testing in Individuals With Low Literacy Levels

In order to evaluate the COVID Radar app as a tool for individuals with low literacy levels specifically, 2 types of activities were carried out by Pharos: the Dutch center of expertise on health disparities. The first activity was an expert review of the self-report monitoring questionnaire by 2 experts from Pharos. All the text was reviewed, considering that the language needed to meet language level A2 or B1 according to the Common European Framework of Reference for Languages [12]. They also made suggestions for adapting the text based on their checklist for accessible information. The checklist contains relevant information related to the accessibility of information in terms of, for example, layout (eg, ensuring there is sufficient color contrast between the text and the background), dosage of information (eg, stating the most important message at the beginning and repeating it), and phrasing and readability of text (eg, avoiding or explaining difficult technical terms or medical jargon). On the basis of the results of the expert review, the researchers updated the phrasing of the questions in the self-report monitoring questionnaire.

The second activity was the usability testing of 4 individuals with low literacy levels. In a one-on-one web-based setting, 4 participants joined a 1-hour usability testing meeting with an expert from Pharos. During this usability testing, the participants were asked to perform assignments to check whether the navigation was simple and logical. They were also asked to read the text aloud so that it became clear which words or sentences were difficult to read. In addition, the participants were asked to explain the meaning of the text in their own words. Finally, the participants were asked for feedback about the entire usability testing process by asking them for alternatives or possible solutions to encountered difficulties. The results of the usability testing were summarized and presented by Pharos to the involved researchers.

### Ethics Approval

Substudies 1 and 2 were declared as not falling under the scope of the Dutch Medical Research Involving Human Subjects Act by the medical ethical committee of the LUMC and were granted a certificate of no objection accordingly. Substudy 3 was certified and performed by a third-party organization (Pharos). All volunteers in these studies signed informed consent forms before their participation.

## Results

### Overview

The codebook developed and used for substudies 1 and 2 is presented in Table 1. The 8 themes were (1) user friendliness, (2) motivation for use, (3) publicity and enlarging the user population, (4) monitoring questionnaire, (5) in-app updates, (6) FAQs, (7) Radar section, and (8) push notifications.

**Table 1:** The codebook used for coding the transcripts of the focus groups (substudy 1) and the email feedback (substudy 2).

Theme and codes		Description	Examples
**Theme 1: general opinion** **of the app**			
	User friendliness	Comments related to the user friendliness of the app; for example, the ease of using the app and navigating through the app	“I find it very down-to-earth, simple, easy to use” “It is a pleasant app to use, but it is indeed very text-heavy. No symbols are used, so you will mainly reach the people with high literacy levels.”
	Design	Comments related to the design of the app, such as colors and layout	“I’m just wondering here, but not all colors may be easy to distinguish on mobile phones, because of the often used colors dark blue, light blue, and gray. On many mobile phones it is not easy to distinguish them.”
Theme 2: motivation for use		Comments related to participants’ motivation and reasons for using the app	“I started using the COVID radar to help you gain insight…” “It is not only an app that is relevant to the field of research but also relevant to oneself. You can also use it to monitor your own behavior and the behavior of your environment. So it also holds up a mirror to oneself.”
Theme 3: publicity of the app and enlarging the user population		Comments related to increasing the user population of the app and supporting the national awareness of the app	“Almost all of us [referring to focus group participants] have a connection with Leiden or the Leiden University Medical Center. I live in Leiden and the only newspaper in which I read something about the app was a local Leiden newspaper, so more national publicity may persuade other people to start using your app.”
**Theme 4: self-report monitoring questionnaire**			
	Content	Comments related to the content of the questions, such as language issues, understandability, suggestions for additional questions, and other suggestions for improvements related to the self-report monitoring questionnaire	“It is impossible to estimate how many people come within five meters when you are at something like a market or a supermarket.” “I think the question is how many hours were you out of the house yesterday. Of course it is clear in itself. But the explanation says that a holiday destination counts as leaving home. Well, at one point we spent a week in a house a while ago...I find that a bit strange that I was out of the house that whole week, 24 hours a day. I don’t think that makes sense.”
	Length and duration	Comments related to the time it took to fill in the questionnaire or about the number of questions	“And what strikes me is that more and more questions are added and I understand that you want to know more about vaccination and everything, but the list is getting very long.”
	Additional information	Comments related to the use of the icons for additional information or elaboration of questions as well as its content	“Yeah in the beginning I clicked on the icons an read their content, you know. Because at one point I wondered ‘yes what about that?’: do I need to count being in the garden as time outside of the house? Well, that didn’t appear to be the case.”
	Frequency of completion	Comments related to the frequency of filling in the questionnaire or about when and where participants completed the questionnaire	“Usually, I fill it in at night when I’m on the couch. Often then I think: ‘oh yeah that radar.’” “Every day! I’m one hundred percent sure. It’s almost something compulsive, that I feel that I need to complete it, I must not forget.”
**Theme 5: in-app updates**			
	Frequency	Comments related to about the frequency or timing for the updates	“I think those updates are great, just like what participant X just said; it could be as often as weekly.”
	Effect	Comments related to the experienced effects of in-app updates	“They mainly motivate me. Then I think, oh well, nice to hear something back. I also think because I have been using the app for more than a year now, I then also see what I am doing it for. So therefore yes I always read the updates.”
	Content	Comments related to the content of the updates	“Generally, I find the updates informative and it’s good to have some more background information about the whole subject.”
**Theme 6: FAQs^a^**			
	Reason(s) for consulting	Comments related to (the) reason(s) of why a user consulted the FAQs section and suggestions for improvement	“Yes, certainly I looked at the FAQ section. Especially in the beginning before I started filling in the questionnaire. Just to see who is behind this app and what they are doing with my data.”
	Content	Comments related to the content of the FAQs section, such as the clarity of answers and the completeness of topics	“This section is clear. Yes, and the answers are too.”
**Theme 7: Radar section**			
	Content	Comments related to the content of the Radar section (eg, maps and sliders) and its effect	“I think you have those sliders regarding behaviour; how many hours have you been outside. It took me a really long time to realize that there are three sliders and not two…”
	Frequency of visiting	Comments related to how often a user takes a look at the Radar section	“Yes, I look at it daily. Or daily, every time I fill in the list, that’s not necessarily daily.”
**Theme 8: push notifications**			
	Suggested improvements	Comments related to suggested ways to improve the push notifications, for example with regard to the timing and frequency	“A personalized pushing message, like for me it is part of my morning routine. When I forget to fill in the questionnaire, I would prefer to hear it [the reminder] in the morning because it happens before nine o’clock or it wouldn’t happen.” “And I think that reminder, whether that is by means of a sound or a pop-up, certainly helps. I have noticed myself… Yes, if I would be able to set a reminder on my own time with a snooze function, then I think the completion score would be much higher.”
	Effect	Comments related to the effect of the (nonintelligent) push messages	“Well I have to say, I’m really happy with those reminders you get. Because, I am someone who does not complete the questionnaire routinely during a particular part of the day, unfortunately. So I have noticed that it helps when I receive such a message, like a push message.” “I turned them off, because I thought it was very mean that I completed it [the questionnaire] and still received a reminder.”

^a^FAQ: frequently asked question.

### Substudy 1: Focus Group Interviews

#### Overview

In all, 2 focus groups comprised 5 participants and a focus group comprised 4 participants because of a participant not being able to join the interview owing to personal circumstances. Of the 14 participants, 12 (86%) were female. Other demographic information was not acquired to preserve the anonymity of the participants. The results of substudy 1 are summarized in Table 2. In the text, a brief explanation of the results is presented, according to the 8 identified themes (Table 1).

**Table 2:** Results of the focus groups as summarized in strengths and potential improvements.

Theme and subthemes			Strengths		Potential improvements
**Theme 1: general opinion** **of the app**					
	User friendliness	Simple and intuitive to use Previous answers being saved as default answers		For some users, the app was a bit too simplistic, which made the app experience boring	
	Design	Basic and simple Satisfaction with functionalities		Create a dark mode option Use of more symbols and less text More use of effective color contrast	
Theme 2: motivation for use			Enables app users to help advance science and contribute to data collection Increased awareness of social distancing behavior		Increased exposure to the research goals and the broader aim of the COVID Radar app Add motivational tools such as gaming elements
Theme 3: publicity of the app and enlarging the user population			High engagement of citizens with a direct or indirect connection to the research organization and its city		More effort in terms of national publicity campaigns to enlarge the national user population More effort in terms of publicity campaigns specifically in geographic areas with relatively low user engagement
**Theme 4: self-report monitoring questionnaire**					
	Content	Most questions were deemed as important and logical by the users		Phrase some questions including their answer categories in a more clear and uniform manner, reducing ambiguity Adapting or deleting difficult-to-answer questions or questions that cannot always be answered in a reliable manner	
	Length and duration	The duration of less than a minute supports regular completion		Create the opportunity for app users to shorten the questionnaire by introducing more logic (ie, skipping questions, where not applicable)	
	Additional information	Overall clear		Elaborate on the provided explanatory information to increase the understanding of questions and thereby provide more support for selecting the right answer category	
	Frequency of completion	Most users completed the questionnaire on a regular basis		Personalized push notifications to motivate to complete the questionnaire and help to create the habit of filling in the questionnaire regularly Also refer to potential improvements for theme 2 (motivation for use) as well as theme 5 (in-app updates)	
**Theme 5: in-app updates**					
	Frequency	None		More frequent updates	
	Effect	Encourages and motivates to complete and continue to complete the questionnaire		None	
	Content	Interesting		Increase the understandability of presented figures	

Theme 6: frequently asked questions—content			Clear and comprehensive		None
**Theme 7: Radar section**					
	Content	Section provides insight in symptoms and behaviors compared with peers		Increase the understandability of the figures by increasing its design and elaborate on and better explain the corresponding functionalities.	
	Frequency of visiting	None		Making the Radar section more visible to users.	
Theme 8: push notifications			Valuable; helpful in completing the questionnaire more frequently		In case of continued use of automated notifications, disable notifications when questionnaire is already completed Enable personalization in terms of frequency and timing of the notifications

#### General Opinion of the App

In general, participants evaluated the COVID Radar app positively. They found the app to be user-friendly and intuitive. Most of the participants were pleased with the design and functionality of the app, with its simplicity frequently being listed as one of its strengths:

I find it very down-to-earth. Simple and easy to use. For me the app is loud and clear, yes, I don’t think it could be more easy.

On the other hand, some participants found the app to be a bit too basic and suggested that the simplicity of the app did not challenge them sufficiently:

How to reach a different or bigger target audience? I think you will have to fix something in the app itself. I am going to say something ugly, but the app looks superficial.

Overall, participants appreciated the fact that their most recent answers regarding COVID-19 symptoms were saved as default answers, thereby saving them time the next time they filled in the questionnaire.

#### Motivation for Use

Participants’ main motivation to use the app can be summarized as their desire to advance science. Participants were motivated to contribute to data collection to help scientists predict future COVID-19 flare-ups:

I’m very pleased with the app, because it gives me the feeling that I’m contributing to something. Of course, that is a great feeling, especially in this time where you often feel helpless.

In addition, several participants mentioned that the time spent reflecting on their social distancing behaviors increased their awareness thereof. For example, when reporting the number of people coming within 1.5 meters or the number of visitors received in one’s home, if these values were relatively high, participants reported experiencing a sort of discomfort that motivated them to reflect on their behavior:

And then you think, well, perhaps I could do that differently next time. It didn't lead me to self-report it differently than it actually was, although I did think from time to time “maybe I should report less people.” But it has kept me on my toes every day, so in that sense I’m definitely doing it for you [the researchers/science], but I’m also doing it for myself and for my own awareness.

When discussing the motivation to keep using the app, several participants discussed the potential of integrating motivational tools in the app to increase adherence. Particular suggestions included repeated exposure to the research goals and the broader aim behind the COVID Radar; for example, by means of in-app updates. Other suggestions related to the potential addition of serious gaming elements, such as enabling participants to collect digital badges based on the number of times they completed the questionnaire:

But you could also use badges, you know. I have another app in which I earn badges for 5 days, 10 days, and 50 days. But then you have to make some kind of dashboard available in the COVID Radar where one can see if one has completed the questionnaire. I know that I am very motivated by those kinds of streaks.

#### Publicity of the App and Enlarging the User Population

Most participants in the focus groups became aware of the app through a direct or indirect personal connection to the LUMC or the city of Leiden. More efforts in terms of national publicity campaigns using social media were suggested to reach more citizens. Another suggestion was to advertise the app in geographic areas with relatively low user engagement using pamphlets or local media.

#### Self-report Monitoring Questionnaire

Most feedback on the self-report monitoring questionnaire concerned the content and phrasing of the questions. Several participants pointed out that some questions left room for interpretation by app users, which may lead to biased answers. More specifically, the question “Yesterday, how many people came within 5 meters of you, outside the house?” received extensive feedback. Most participants considered this question challenging or even too difficult to answer, as they did not feel capable of accurately estimating this number:

I ride my bike almost daily. How many people do you come across within 5 meters? A street is 5 meters, so every time I ride my bike I am almost sure that I can fill in that 50 people came closer than 5 meters to me.

The question “Are you completely vaccinated against COVID-19?” also received extensive feedback. Some participants wondered why there was only an interest in whether they were completely vaccinated, instead of allowing users to indicate how many times they had received vaccination.

Regarding the additional information accompanying the questions, the provided information did not always clarify questions or doubts the participants had concerning the respective question. For example, there were numerous questions regarding whether certain locations, such as work offices, shops, and supermarkets, should be categorized as public places.

Concerning the length of the questionnaire, some participants found it too long, although most participants reported that filling in the questionnaire took less than a minute. Relatedly, some participants struggled with their motivation to answer the same questions repeatedly. One suggestion was to enable the possibility of indicating whether the participant experienced changes in COVID symptoms, as these were found to be relatively steady in comparison with behavioral measures (eg, one does not have a sore throat every other day, whereas the number of people coming closer than 5 meters can fluctuate by day). More specifically, participants suggested creating a shortened version of the questionnaire in which the symptom complaints of COVID-19 were combined into one question (eg, *“Are you experiencing any COVID symptoms?”*) which, if answered positively, resulted in a pull-down menu in which they could indicate which specific symptoms were present.

Most participants reported completing the monitoring questionnaire regularly, with some reporting doing so daily. Most participants reported having made a habit of filling in the questionnaire at a set time each day:

It needs to become just like brushing your teeth.

Participants who filled in the questionnaire on a less regular basis seemed to report less consistency in their timing of completion, struggling to create a habit.

#### In-App Updates

Most participants read the updates as soon as they noticed the in-app notification, whereas others never received or noticed any notifications and checked this section once in a while for updates. Overall, the push messages of the in-app updates were evaluated as being valuable.

Participants were enthusiastic about the content of the updates and described the updates as very interesting. Furthermore, participants elaborated on how these updates motivated them to keep filling in the questionnaire:

For me, the updates are the most fun part of the app, because it is some kind of reward for completing the questionnaire.

However, numerous participants mentioned that the figures used in the updates were not always comprehensive or explained in sufficient detail. For example, the color scheme was a source of confusion, with some participants not being able to fully understand the figures. It was also mentioned that the axis labels were sometimes lacking or incomplete. Almost all participants indicated that they preferred more frequent updates.

#### Frequently Asked Questions

Approximately half of the participants reported having accessed the FAQs section of the app at least once. Reasons for consulting the FAQs section ranged from being interested in the research and corresponding analyses performed with the collected data and wanting to know more about privacy-related issues to curiosity as to how many active app users there were. In general, participants perceived the information in this section to be clear and comprehensible.

#### Radar Section

For a few participants, the Radar section of the app provided a convenient method for comparing their symptoms and behaviors with those of their peers. They elaborated that they sometimes adjusted their behavior based on peer comparison data:

I compare myself to other people in my region. I am sensitive to social pressure, so for me sometimes this is a reason to hold myself back on the weekends.

Nevertheless, the Radar section received the most attention in terms of identified optimization targets. Two participants had never noticed the Radar. The visuals (ie, figures) presented in the Radar section were another frequently mentioned subject of potential improvement. Lack of contrast and nonmatching colors in the legends and figures were described as hindering to understanding by the app users.

#### Push Notifications

Participants who did not complete the questionnaire daily reported that the push messages motivated them to completing the questionnaire more frequently:

I am very pleased with the reminders… The reminders definitely encourage me to fill in the questionnaire.

In contrast, participants who completed the questionnaire daily reported that the push messages created confusion. The messages made them question whether or not they had already completed the questionnaire on that day. They also reported the reminders to be a source of irritation when they were certain they had already completed the questionnaire that day. A potential solution offered by the participants was to create the possibility of personalizing push notifications, allowing them to be set at a user-defined time and frequency, thereby helping them to create a routine.

### Substudy 2: Mailbox Analysis

A total of 1080 emails were analyzed; approximately one-third of the emails were coded as irrelevant, as they comprised either duplicate mail, spam mail, mail commercials, or updates from the COVID Radar Facebook or Instagram accounts. Most emails contained questions from app users on how to interpret or answer a specific question from the self-report questionnaire (Table 1, Theme 4: content). The monitoring question about how many people came within 5 meters on a particular day resulted in extensive feedback from many users, indicating that they felt it was not reasonable to accurately estimate this value. In addition, users asked several questions about which contacts to count as “people coming within 5 meters”:

On my way to work I pass many people that come within 5 meters of me. Should I report all these brief contacts in the Radar?

Another monitoring question that received extensive feedback pertained to the question about the number of visitors one had received at home during a particular day. Users often questioned whether they needed to count their grandchildren aged <12 years as visitors:

We take care of our grandchildren. Of course, they do come closer to me than 1.5 meters, but we have understood that the risk of infection via children is minimal. Do we need to report our grandchildren as people we came closer than 1.5 meters?

Several emails contained suggestions for additional questions to be included in the monitoring questionnaire. For example, many individuals suggested adding a question on vaccine status once the vaccination program started in January 2021. Another common point of feedback was that the answer format of the self-report questionnaire did not allow for any nuance. Some users reported feeling badly about their social distancing habits, for example, owing to their role as caregivers for older persons or sick family members:

I’m sure it sometimes looks like as if I don’t stick with the rules. But if the questions would allow for any nuance, especially regarding the age of visitors and informal care of, for instance, older family members, it would be a totally different outcome for me.

Furthermore, numerous emails contained feedback on the Radar section of the app (Table 1, Theme 7: Radar section). These primarily consisted of questions on how to adequately interpret the data in the maps, as the colors in the maps did not always correspond to those in the legend. In addition, numerous emails contained comments about the answer scales of the questions regarding social distancing behavior, which were originally formatted as sliders, resulting in negative feedback from users who found the sliders difficult to use, often resulting in reporting an incorrect number of contacts. On the basis of this feedback, the sliders were replaced with a box in which the number of contacts can be entered manually, and (+) and (–) buttons can be used to manually adjust the number of contacts. This resulted in numerous emails receiving positive feedback.

### Substudy 3: Usability Testing in Individuals With Low Literacy Levels

On the basis of input from experts from the Dutch center of expertise on health disparities, and usability test sessions with individuals with low literacy levels, several optimization targets were identified regarding the design of the app. One related to improving the use of colors and color contrast to improve the visibility of letters. For example, the blue color used to color the boxes of default answers in the self-report monitoring questionnaire (Figure 1) provided limited color contrast with the white letters, sometimes leading to the default answer options being overlooked by users. Further optimization targets regarding design were to highlight the differences in the answer options by using bold text or underlining, as users with low literacy levels may otherwise have difficulty in distinguishing between slightly different answer options. A participant in one of the usability test sessions indicated the following:

I think it says the same thing twice, I don’t see what the difference is.

Other outputs regarding design optimization were to instruct end users more explicitly on all app features. For example, that they need to scroll down for more questions, and that they need to press “submit” after completing the last question.

Regarding textual optimization, adding information was sometimes suggested to improve comprehension of questions. For example, it was suggested we define “fever” as not everyone is aware that this is defined by a body temperature of ≥38 °C. Further, several simplifications of the text were suggested, such as to simplify or elaborate on “moderate to severe psychical exercise.” Other textual suggestions included writing out “one-and-a-half meter,” as 1.5 meters is often read as “1 to 5 meters” by individuals with low literacy.

## Discussion

### Principal Findings

A total of 3 qualitative substudies were conducted, and numerous optimization targets for the COVID Radar app were identified, both in terms of the design and content of the app. Convenience of app use, personal adjustments of the app experience, and motivation to keep using the app are key to procuring and maintaining a population of active users engaging with public health surveillance apps such as the COVID Radar. This information can be used to maximize user satisfaction, adherence, and engagement with national surveillance data collection apps.

Optimization targets as identified by the 3 studies included highlighted design elements, such as the importance of using sufficient color contrasts as well as using more visuals and less text to increase the user friendliness of the app and the comprehensibility of the corresponding texts. The results of substudy 3 highlight the importance of increasing the accessibility and comprehensibility of text for individuals with low (health) literacy levels, for example, by using language level A2 or B1 according to the Common European Framework of Reference for Languages [12] and by conducting usability tests with this target population. Limited (health) literacy levels are a prevalent and common concern worldwide [13-15]. It is essential that public health services and tools simplify their use and content to address health disparities and increase the inclusiveness of such services. High user engagement and adherence are crucial to the success of web-based monitoring tools for population-based surveillance and corresponding successful predictive modeling [3,16].

Although the feasibility and acceptability of using smartphone apps for continuous monitoring of disease seems evident from the literature [17-20], motivating individuals to use and especially keep using such tools remains challenging. The results of substudy 1 showed that the main motivations of COVID Radar users engagement were contributing to scientific research and, more specifically, helping fight the pandemic. This is in line with the available literature in the field of citizen science demonstrating that participants in citizen science projects are motivated to contribute to the scientific goals of such projects [21]. Furthermore, these results are in line with 2 important categories of community involvement in scientific projects identified by Batson et al [22]: altruism and collectivism. That is, motivation to be involved in increasing the welfare of another individual and the welfare of a group, respectively. Along similar lines, the results of substudy 1 showed that app users were eager to know what was done with their data and that in-app updates informing them about recent analyses using COVID Radar data were an important source of motivation to keep using the app. This is in line with the results of a citizen project by Land-Zandstra et al [21], who showed that participants wanted to be kept informed about the project and its outputs. Our findings also confirm that highlighting data use can be an effective means of supporting individuals to participate in citizen science projects, as shown by Rotman et al [23].

Another potential strategy to increase user engagement and motivation is the incorporation of persuasive technology such as gamification: the use of gaming elements such as challenges and rewards to a nongame environment. Although still in its early stages, preliminary evidence supports the use of gamification to increase use and user engagement in internet interventions focusing on (mental) health and behavior change [24]. However, the effects of gamification on initiation, adherence, and engagement in a long-term monitoring population-based surveillance app remain unclear. Finally, the results from the focus group interviews demonstrated that convenience of app use (ie, user friendliness and limited time needed to complete the monitoring questionnaire) and enabling personalization of the app (ie, personal settings in terms of colors, layout, and reminders) are other important factors to consider when attempting to maximize user engagement and motivation.

### Limitations

The main limitations of substudies 1 and 2 pertain to sample bias; it is likely that the participants in the focus groups were above-mean motivated app users with more positive attitudes toward the project and the app. Likewise, email feedback is likely to be sent more frequently by highly engaged app users, whereas less motivated users or users with negative evaluations or experiences with the app may have stopped using the app and were thus not involved and reached by substudies 1 and 2. Another potential limitation is the selection bias based on the online nature of the interviews: because the focus groups were conducted using Zoom, possible participants who were not yet familiar with this program might have decided against participation for this reason. To reduce this bias, we offered a practice round to participants who were not yet experienced in using Zoom. One participant took advantage of this opportunity. NS was available for technical assistance, which was not requested during the 3 focus group interviews. However, we cannot claim that other potential participants with a lower digital literacy were not deterred from participation. However, the participants in substudy 3 had not previously used the app; therefore, their feedback on the textual features of the app was unbiased by previous experience with the app. Furthermore, the results of substudies 1 and 3 may be limited in terms of generalizability because of the relatively low number of participants in these substudies (N=14 and N=4, respectively). The use of web-based interviewing further comprised the ability to fully assess body language. However, the interviews were conducted via videoconference, so facial expressions could be read and interpreted by the interviewer. Therefore, it is not believed that this biased our conclusions regarding the focus groups.

### Conclusions

A self-report population-based surveillance mobile app seems useful in national research on symptoms and social distancing behavior. The main motivations of citizens to use such an app are related to altruism, collectivism, and wanting to help science in developing and improving mobile self-report apps in the context of public health surveillance tools. Convenience of app use, enablement of personalization of the app, and motivational factors are key to procuring and maintaining a population of active users engaging with an app such as the COVID Radar. When designing such an app and developing content, it is important to consider its accessibility for individuals with low literacy levels. These results are not only critical to the optimization of the COVID Radar app, ultimately increasing its acceptability, inclusiveness, and adherence, but are also relevant in the broader context of citizen science projects and public health surveillance tools during the pandemic.

## Appendix

Multimedia Appendix 1 Final and full versions of the COVID Radar self-report questionnaire.

## Abbreviations

FAQ: frequently asked question
LUMC: Leiden University Medical Center
