# Supplementary material for: Optimizing the Acceptability, Adherence, and Inclusiveness of the COVID Radar Surveillance App: Qualitative Study Using Focus Groups, Thematic Content Analysis, and Usability Testing
Source: JMIR Form Res. 2022 Sep 9;6(9):e36003. doi: 10.2196/36003 (PMC9466658; doi:10.2196/36003)
Supplement: Multimedia Appendix 1 [file formative_v6i9e36003_app1.docx]

## Multimedia Appendix 2: The current and full version of the COVID radar self-report questionnaire

| **Question** | **Answers** | **Additional information** |
| --- | --- | --- |
| Have you come within 1.5 meters of a Corona patient in the last 14 days? | * No  * Yes | A person is a "corona patient" if he or she has tested positive or if a doctor has expressed strong suspicion of corona infection. Otherwise, the answer is "NO". The answer is also "NO" if that person has not shown any symptoms during the past 24 hours (fever, cold nose, cough) and is therefore no longer contagious. |
| Do you have a cough? | * No  * Yes |  |
| Do you have a throat ache? | * No  * Yes |  |
| Do you have a fever(38 degrees or higher)? | * No  * Yes |  |
| Are you short of breath? | * No  * Yes |  |
| Do you experience (stinging) pains in your chest? | * No  * Yes |  |
| Are you experiencing brain fog? | * No  * Yes |  |
| Are you experiencing sadness or depression? | * No  * Yes |  |
| Are your taste or smell affected? | * No  * Yes |  |

| **Question** | **Answers** | **Additional information** |
| --- | --- | --- |
| Do you suffer from fatigue? | * No  * Mild fatigue  * Severe fatigue |  |
| Do you have a headache? | * No  * Yes |  |
| Have you had the corona virus (confirmed via a test done by the GGD or your GP)? | * No  * Yes |  |
| Have you been tested in the past two weeks for the corona virus? If so, what was the result? | * No – have not been tested  * Have been tested – result was that I did not have the corona virus  * Have been tested – result was that I did have the corona virus | If you have been tested multiple times in the past two weeks, fill your answer in for the most recent test. |
| Are you completely vaccinated against COVID-19? | * No  * Yes | Most vaccines require two doses to be completely vaccinated. If you received the Johnson & Johnson (Janssen) vaccine, only one dose is needed. Your GP or a GGD associate can help answer any questions you may have on vaccination. |
| Did you leave the house yesterday to go to work/school? | * No  * Yes | This is only about these two reasons to leave the house. Thus, hiking and grocery shopping are not included, they are included in the next question. |
| For how many hours were you outside the house yesterday? | Numeric scale | Your garden is considered "at home". Holiday destinations are considered "outside". |
| Yesterday, how many people came within 5 meters of you, outside the house? | * None  * 1-10  * 11-30  * 31-50  * More than 50 |  |
| **Question** | **Answers** | **Additional information** |
| How many visitors visited your house yesterday? | Numeric scale | Visitors in the garden are considered visitors to the house. Children count as well. |
| Yesterday, how many of the people in the two questions above, came within 1.5m of you? | Numeric scale | Children and contacts where you or the other person wore a non-medical mask count |
| Did you sport for more than 30 minutes, yesterday? | * No  * Yes, mostly inside at a sports club or event  * Yes, mostly outside at a sports club or event  * Yes, somewhere else (e.g., at home, in a park, etc) | We consider medium to heavy exercise (e.g. hiking, cycling, running or playing soccer) and muscle or bone strengthening exercise (like strength or balance exercise) sports |
| Did you wear a face mask yesterday, regardless whether or not those around you wore one? | * No or not applicable  * Yes, more than what is legally required (more often or better mask)  * Yes, only as legally required | A mask is required on public transport and at Schiphol airport. |
| Are you currently abroad? | * No  * Yes, outside of Europe  * Yes, inside of Europe (choose country from pulldown menu) |  |
